# [Retracted] *Tremella* polysaccharides inhibit cellular apoptosis and autophagy induced by *Pseudomonas aeruginosa* lipopolysaccharide in A549 cells through sirtuin 1 activation

**DOI:** 10.3892/ol.2024.14384

**Published:** 2024-04-08

**Authors:** Xiaolan Shi, Wenfeng Wei, Ning Wang

Oncol Lett 15: 9609–9616, 2018; DOI: 10.3892/ol.2018.8554

Following the publication of this paper, it was drawn to the Editor's attention by a concerned reader that the flow cytometric assay data in Fig. 2A and the Hoechst 33258-stained images in Fig. 2B on p. 9612, and the western blotting data shown in Fig. 4D on p. 9614 had already appeared in previously published articles written by different authors at different research institutes. Owing to the fact that the contentious data in the above article had already been published prior to its submission to *Oncology Letters*, the Editor has decided that this paper should be retracted from the Journal. After having been in contact with the authors, they accepted the decision to retract the paper. The Editor apologizes to the readership for any inconvenience caused.

